# Advanced Laguerre Tessellation for the Reconstruction of Ceramic Foams and Prediction of Transport Properties

**DOI:** 10.3390/ma12071137

**Published:** 2019-04-08

**Authors:** Christos S. Stiapis, Eugene D. Skouras, Vasilis N. Burganos

**Affiliations:** 1 Institute of Chemical Engineering Sciences (ICE-HT), Foundation for Research and Technology, Hellas (FORTH), Stadiou, Platani, GR-26504 Patras, Greece; christosstiapis@iceht.forth.gr (C.S.S.); eugene.skouras@iceht.forth.gr (E.D.S.); 2 Department of Chemical Engineering, University of Patras, GR-26504 Patras, Greece; 3 Department of Mechanical Engineering TE, TEI of Western Greece, GR-26334 Patras, Greece

**Keywords:** ceramic foams, reconstruction, porosity, tortuosity, permeability, Laguerre tessellations

## Abstract

Ceramic foams are promising, highly porous materials, with a wide range of specific surface area and low fluid flow resistance, which are well-suited for filtering applications. They are comprised mainly of macrovoids that are interconnected with struts. A branch-shaped reconstruction algorithm is introduced in the present work to reconstruct various ceramic foams from electron microscopy images using the Laguerre tessellation method. Subsequently, the reconstructed samples are used for the numerical calculation of pore structure and transport properties, including specific surface area, tortuosity, effective diffusivity, and flow permeability. Following comparison with experimental data, this reconstruction method is shown to be more reliable than typical analytical expressions that are suggested in the literature for the aforementioned structural and transport properties. Extracting the equivalent pore radius of the reconstructed domains offers improved accuracy of the analytical expressions for the permeability estimation.

## 1. Introduction

Ceramic foams are highly porous materials that are utilized in a wide spectrum of various technological applications [1,2]. They are mainly produced by a polymeric sponge method. In this process, a polymeric foam is used as a template and is immersed in a ceramic slurry, where it is subsequently dried and sintered, yielding a close replica of the original polymeric foam [3]. Normally, the structural properties of the ceramic filter produced by this technique are mostly dictated by the characteristics of the polymer foam, such as the porosity, the pore size, and the number of pores per linear inch (PPI) [4,5]. Thus, characterization of the foams is crucial for the determination of their suitability under specific operational conditions, including the estimation of significant properties, such as flow, and chemical and mechanical resistances [6,7,8]. The permeability is an important characteristic of ceramic foams that is mainly useful in filtration processes. An accurate estimation of the pressure drop across the filter allows control of the energy that is required for the flow to overcome the pressure gradient [9]. Numerous efforts have been made to estimate the permeability of porous media that initially relied on experimental fitting [10], and later resulted in analytical models with specific and well-defined, yet simple, topologies [11,12,13,14]. Those analytical expressions are valid only for a specific range of porosities, as well as for a specific range of PPIs, with the foam topology usually being treated as an ordered arrangement of void cavities (pores) and solid, interconnected branches. However, the main challenge in the permeability evaluation using the aforementioned analytical expressions is the accurate definition of key structural properties of the cellular medium in order to supersede the pore diameter, *d*_p_. Predominantly, it is difficult to provide a global definition for the pore size, due to the fact that pores are characterized by a distribution of sizes and a variety of shapes. Two exceptions to this rule arise in the special cases of either non-overlapping cylindrical pores, or non-overlapping spherical pores, both with uniform size. In contrast, with granular media that are characterized by a mean particle size, difficulties arise in the definition of a single size for pores in the case of foam structures, which can be practically viewed as solid branches or solid items interconnected in three dimensions. However, the ability to predict the permeability of a ceramic foam with non-invasive methods remains highly important, especially in cases where the experimental procedure must be conducted in strenuous environments, such as in the flow of molten metals [9].

A popular approach is to treat the foam itself as a modular system reconstructed by spatial tessellation [15]. The tessellation methods used in the literature can be either stochastic [16] or deterministic in nature [17]. The tessellations that are obtained by deterministic approaches, which focus on replicating a single type of polyhedron, usually ignore the variation and the anisotropy of real foams [18]. The stochastic tessellation methods found in the literature are heavily based on the Poisson–Voronoi algorithm (PVA) [19]. In these methods, a Voronoi tessellation is performed on a point cloud generated by a Poisson process. However, certain important geometrical properties of the generated foams obtained by the PVA method fail to compare satisfactorily with the corresponding properties of the real foam. More specifically, the average number of cell faces per generated cell has proved to be higher than that in the real foams [18], and the cell sizes follow typically a lognormal distribution [20]. An alternative approach was adopted by Lautensack et al. [21], who used the Laguerre tessellation to describe the foam morphology. Comparison with the PVA and the Hard Core Voronoi methods shows that the Laguerre tessellations are very promising for the representation of the foam topology. Moreover, Randrianalisoa et al. [22] utilized Laguerre tessellations for the generation of open-cell foams and focused on heat conduction—assumed a circular cross-section shape for the edges—and obtained promising results. Although these methods use a tessellation process, they do not warrant a cell size similarity to the actual foam, much less a branch shape similarity between the reconstructed foam branches and the branches observed on scanning electron microscopy (SEM) images.

The main scope of this work is to study the transport properties of a number of characteristic ceramic foams in all three Cartesian directions, by establishing a digital reconstruction procedure. For validation, as well as for characterization purposes, a comprehensive set of calculations is conducted to obtain the mean pore diameter, the tortuosity factor, the pore size distribution (PSD), and the effective permeability for the digitally reconstructed model of each foam, followed by comparison with data from the literature. Using the proposed methodology, three different foams are reconstructed in order to show the range of the applicability of the methodology to model different types of ceramic foams of various PPI. In the present approach, a Laguerre tessellation method is developed for the reconstruction of ceramic foams consisting of convex cells with a large variance of cell sizes. The SEM images of the foams are used for the extraction of structural information on certain special features of the foam under consideration. A procedure to sample the cell size and the branch shape directly from the SEM images is presented and offers data that are utilized in the process of foam reconstruction.

## 2. Materials and Methods

The most widely used methods for the reconstruction of foamy domains are tessellations. Among the tessellation methods, the three most well-known and popular ones are the Voronoi tessellations, the Laguerre tessellations (also known as power diagrams), and the Johnson–Mehl tessellations. All three models are based on the isotropic growth model. The Voronoi tessellation builds on a random N-dimensional point distribution, and creates convex hulls generated by an isotropic growth starting from the point germs [23]. The Laguerre tessellations operate in a similar fashion to the Voronoi ones. The main difference lies with the radius/power assigned to each point of the random process. In the present work, the Laguerre tessellation option was used in order to obtain an arbitrary foam-like structure. The polyhedral cells that are formed during this process are assumed to represent the void space of the membrane. On the contrary, polyhedral edges are fabricated with branches that represent the pore wall domain. The mathematical description is a tessellation generated by a locally finite set of weighted points called nuclei, generated by an appropriate point process and interacting through the power distance that forms polyhedral cells:

(1) $$\left[\vec{x},w\right]\in \psi \subset {\mathbb{R}}^{n}\times \mathbb{R},$$

(2) $$pow\left(z,\left[\vec{x},w\right]\right)=\left\|z-\vec{x}\right\|-w,$$

(3) $$Cell\left(\left[\vec{x},w\right],\psi \right)=\left\{z\in {\mathbb{R}}^{n}\|pow\left(z,\left[{\vec{x}}^{*},w^{*}\right]\right),\left[{\vec{x}}^{*},w^{*}\right]\in \psi \right\}.$$

The mathematical description can be interpreted as a sphere, the center of which is located at *x* and has a radius of r_sph_ = $\sqrt{w}$.

In the proposed approach, we assume that the nuclei to be utilized are packings of non-overlapping spheres whose radii are sampled from a prescribed distribution, as extracted from the corresponding SEM image. The main concept makes use of the sphere arrangement in order to approach qualitatively certain geometric characteristics of the foam with the cells produced by the Laguerre tessellation. Thus, we can assume that the tessellations are performed on the set of spheres with a predetermined size distribution. The deposition of the spheres/nuclei was performed with the Lubachevsky–Stillinger packing algorithm [24,25]. This sphere-packing algorithm creates a non-overlapping arrangement of adjacent spheres, and provides promising dynamics during the reconstruction: (i) By principle, the cell that is created completely surrounds the sphere that acted as a seed, thus the minimal cell volume is at least equal to, or larger than, the seeding sphere that generated it; (ii) The size distribution of the tessellated cells is strongly dependent on the distribution and size of the seeding spheres.

At the limit of densely packed spheres, the cells obtained by the Laguerre tessellation were very similar to the size distribution of the seeding spheres. In the latter case, we can claim that a qualitative description of the domain was achieved. Another critical geometrical element of the foam structure was related to the shape of the branch. The majority of the models found in the literature assume branches of a constant diameter along the convex cell edges, resembling the shape of a cylinder. In the present approach, the branch has a more complex axisymmetrical shape, assuming varying thickness along the branch axis. This variation has been shown to affect many of the elastic, thermal, flow, and transport properties of the foam [26,27,28]. The foam micro-structure is intricate and shows many local geometrical irregularities that cannot be reproduced in a deterministic manner, since they are local features of the domain under consideration. However, the overall effect of those geometrical irregularities on the transport properties can be averaged by considering a representative elementary volume (REV) sample of ample size, i.e., at least three times the size of the average pore. In this manner, their impact on the overall transport properties becomes negligible in view of the global morphology of the structure. Evidently, the influence of the micro-structure is of crucial importance for the appropriate description of the fluid flow and, thus, of the transport properties. However, the effect of the local irregularities is, once again, negligible in relation to the overall effects induced by the overall geometry. Thus, the precise description of nodes and struts/branches is crucial for the generation of a representative foam structure, which reproduces the influence of the geometry on the actual transport phenomena that take place in the material. Consequently, this feature was deemed important and necessary to be included in the present model.

### 2.1. Reconstruction

The central goal of the reconstruction process was to obtain the cell skeletons. The Laguerre tessellation procedure was exploited for the generation of the foam bare structure, since it decomposes the original space into an arrangement of space-filling polyhedrons that do not overlap with each other. However, a typical feature of open foams, which is often overlooked, is the fact that the strut thickness varies locally. Normally, the branches become thicker at the ends than at their centers [29]. Moreover, experiments show that the ligament shape is related to the void fraction of the foam [28]. More specifically, the cross-sectional shape of the branch shifts from a circular shape, found at lower porosity foams, to a triangular type, typically at elevated porosities. In this paper, we focused on foam materials that can be approximated by strut cross-sections of a nearly circular form and have a moderate porosity value. The intention was to encapsulate this local strut variation in the model, which was achieved initially by sampling the branch shape from a predetermined distribution, then allocating the newly sampled branch to the corresponding position.

#### 2.1.1. Branch Sampling

**Figure 1:**
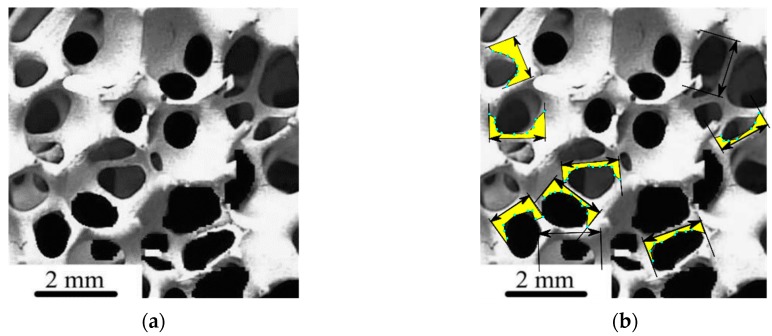
(**a**) The original foam structure (with permission from [30]); (**b**) branch radius profiles (in yellow) along the length of characteristic branches.

The shape profile of the branches was sampled directly from the SEM image (see, for instance, Figure 1a [30]). This was done by considering multiple points on the branch void interphase along the length of each branch (Figure 1b).

By repeating the sampling procedure over all branches on the SEM image, the *i*-th observed branch length, $l_{obs,i}$, defined as the Euclidean distance between the centers of the nodes that are connected by the branch, as well as the cross-sectional radius distribution along the normalized length of the *i*-th branch, were stored in an index list.

#### 2.1.2. Branch Sampling

**Figure 2:**
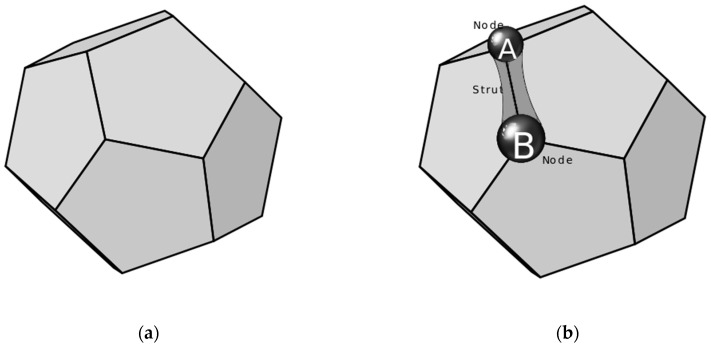
(**a**) A cell obtained from the tessellation representing a polyhedron, stored as a list of interconnected nodes; (**b**) two sequential nodes on a cell, connected by a branch.

The cells obtained from the tessellation represent polyhedrons that are stored in a list of interconnecting nodes (Figure 2a). Thus, in order to generate any two sequentially connected nodes—A with position vector ${\vec{x}}_{A}$, and B with position ${\vec{x}}_{B}$ (Figure 2b)—and handle them as branch ends, the relative position vector connecting the two nodes ${\vec{r}}_{\mathrm{AB}}={\vec{x}}_{B}-{\vec{x}}_{A}$ was calculated and indexed appropriately. The norm of the relative position vector, $l_{\mathrm{AB}}$, is the length of the branch, whereas the direction of the branch is found by the relative position vector. Thus, the unit vector that indicates the direction of the vector connecting the nodes A and B is denoted by dir⇀AB=r⇀AB lAB.

At this point a similarity function was introduced, shown in Equation (4):

(4) $$Sim\left(l_{\mathrm{AB}},l_{\mathrm{obs},i}\right)=\frac{1}{\left|l_{\mathrm{AB}}-l_{\mathrm{obs},i}\right|}.$$

The similarity function is an indicator of the resemblance of the length value for the *i*-th observed branch from the SEM image to the length of the branch connecting nodes A,B. The similarity function obtains higher values for similar lengths and lower values for dissimilar lengths. By obtaining the similarity value between all observed branches and the arbitrary branch connecting A and B, a list of similarity values is generated:

(5) $$SimList=\left\{Sim\left(l_{\mathrm{AB}},l_{\mathrm{obs},1}\right)\ldots Sim\left(l_{\mathrm{AB}},l_{\mathrm{obs},i}\right)\right\}.$$

Finally, the corresponding branch profile was obtained by Tower-sampling [31] the index list with probability proportional to the values of the SimList. Thus, the branch profile was sampled based on the similarity of the length of the generated branch with the length of the sampled branches.

#### 2.1.3. Branch Generation and Allocation

**Figure 3:**
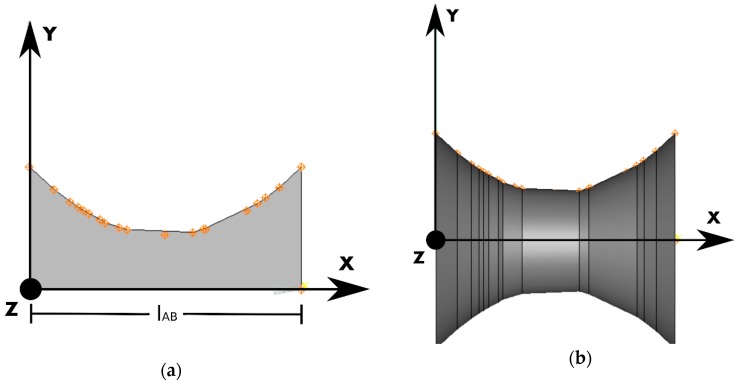
(**a**) A cross-sectional radius distribution of a SEM-measured branch. (**b**) The revolved surface that represents the surface of the branch.

**Figure 4:**
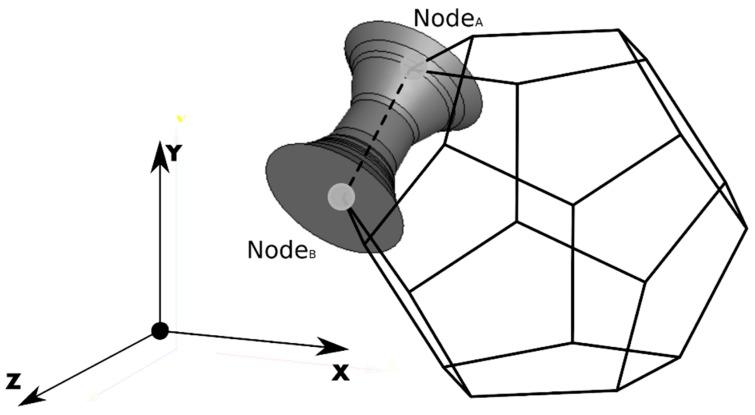
An example of the branch radius profile along the length of the branch.

Each branch had its external surface defined by revolving a planar profile, as extracted from the cross-sectional radius distribution of branches measured on the SEM images (Figure 1b), around the branch axis (Figure 2). The rotation of this planar shape about the branch axis traces the surface by revolution (Figure 3a,b), which defines the shape of the branch. The objective surface that represents the branch after the revolution is performed along the *x*-axis with a unit vector that denotes the orientation of the branch, ${\vec{dir}}_{1}=\left[1,0,0\right]$, is shown in Figure 4.

The next step was the branch placement, which involved translation and orientation of the newly generated branch to align itself with the edge between the nodes, ${\vec{dir}}_{AB}$, shown in Figure 2b. The rotation matrix, R, that rotates the unit vector, ${\vec{dir}}_{1},$ onto the unit vector, ${\vec{dir}}_{AB},$ is calculated as follows:

- Let ${\vec{\nu }}_{1,\mathrm{AB}}={\vec{dir}}_{1}\times {\vec{dir}}_{\mathrm{AB}}$;
- Let $s_{\epsilon }=\left\|{\vec{\nu }}_{1,\mathrm{AB}}\right\|$;
- Let $c={\vec{dir}}_{1}\cdot {\vec{dir}}_{\mathrm{AB}}$.

The rotation matrix is defined by

(6) $$R={\left[1\right]}_{m}+{\left[{\vec{\nu }}_{1,\mathrm{AB}}\right]}_{x}+{\left[{\vec{\nu }}_{1,\mathrm{AB}}\right]}_{x}^{2}\left(\frac{1-c}{s_{\epsilon }^{2}}\right),$$

where ${\left[1\right]}_{m}$ is the identity matrix and ${\left[{\vec{v}}_{1,\mathrm{AB}}\right]}_{x}$ is the skew-symmetric cross-product matrix of ${\vec{v}}_{1,AB}$. Then, the rotated branch was translated in order to obtain its final position in the tessellation skeleton, connecting nodes A and B, as shown in Figure 4.

### 2.2. Determination of the Sphere Size Distribution

The aim of the present methodology is the digital reconstruction of foams, based on the characteristic dimensions of both the solid and the void phase. The pore or cell diameters can be employed for the void phase characterization. The diameters of the pores and the throats (pore windows) were straightforwardly derived from the PPI count, and can be used to approximate a branch length in a cell representation of the solid matrix [32], an action already performed during the branch profile extraction. However, to the authors’ knowledge, there is no well-established method for foams yet that could extract the cell sizes or the equivalent cell diameters of the foam from a SEM image. The quantification of the cell diameter was accomplished here by utilizing ImageJ^®^ software to measure the micro-structural features. In order to threshold the foam images, a newly developed method for image segmentation using mathematical morphology was utilized [33]. This method is based on two tools—the watershed transform and the homotopy modification—which solve the problem of over-segmentation and introduce the notion of markers of the objects to be segmented in the image. Further details on the procedure can be found in the work of Meyer et al. [33]. In the present case, morphological segmentation was performed using an ImageJ/Fiji plugin that combines morphological operations—such as extended minima and morphological gradients—with watershed flooding algorithms to segment grayscale images of any type (8, 16, and 32-bit) in 2D and 3D.

**Figure 5:**
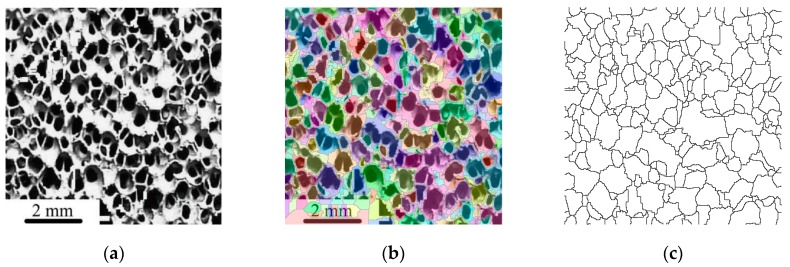
The three steps during cell size extraction from a foam: (**a**) Original foam image (with permission from [30]); (**b**) overlaid image after the morphological segmentation of the original image; (**c**) the segmented binary image where the black lines denote the cell boundaries.

The original foam image of 45 PPI foam is shown in Figure 5a (from [30]). In Figure 5b, the colored object denoting the individual cells is overlaid on the input image. Finally, Figure 5c shows the binary thresholded image, denoting the watershed lines in black and the cell objects in white.

### 2.3. Pore and Throat Size Distributions

The pore size distribution (PSD) is usually considered a more significant structural parameter than the total porosity [34]. The most popular experimental techniques employed in the literature to characterize the pore size distribution are mercury intrusion porosimetry (MIP) [35] and capillary flow porometry [36]. In the present approach, the pore and throat size distributions were extracted by the morphological processing of the three-dimensional images obtained from the reconstruction process. In short, the pore–throat detection algorithm contains the following steps:

- A Euclidean distance transform (EDT) is operated on the three-dimensional image matrix. In this case, for each voxel belonging to the void phase, the maximum inscribed sphere radius from the center of the voxel, i.e., the one that touches the solid phase at one point, is assigned to each void voxel.
- For each void voxel, all the previously generated inscribed spheres containing it in full are then indexed. A void voxel is potentially contained in several inscribed spheres from adjacent void voxels. The center and radius of the largest inscribed sphere that fully contains the voxel (the maximum containing sphere, as defined in Dong et al. [37]) is then assigned to this particular voxel.
- Chamber pores are identified. The previous step generates a 3D map of the maximum encompassing sphere radius for each voxel, and the pore chamber attribute is given to the largest contiguous group of voxels with this sphere center and radius. The center of the maximum encompassing sphere radius is assigned as the pore (chamber) center.
- Pore propagation is performed through the encompassing sphere radii originating from the central pore voxels, to map the boundary voxels of each chamber-type pore. For each pore seed, an iterative process that expands the pore boundaries is performed. At each iteration, the 26 closest neighbors of the pore seed voxel are checked and if a neighbor (i) is void, (ii) doesn’t belong to the current pore, or (iii) its maximum inscribed radius is not larger than the maximum inscribed radius of a current boundary voxel, then this neighbor is identified as belonging to the current pore and is included in the list of boundary voxels for the next iteration. Voxels that form the boundary at any current iteration step are considered as boundaries of the pore for the next iteration. The iterations are terminated when the list of boundary voxels is emptied. However, when this iterative process is ended, each voxel can belong to multiple pores—in this case, the shared voxels are treated as pore throats, i.e., as void space connecting the chamber-type pores.
- Watershed segmentation is performed at overlapping chamber-type pore regions. In 3D, the overlapping volume of adjacent spherical pores are larger and often comparable to the individual pores, a property that is often not desirable. As a further issue, this property increases the coordination number of the pores artificially, due to the fact that pores spread largely and have a lot of connections with other pores. A remedy to the problem of having too large coordination numbers is the modification of the algorithm to produce single-pixel thick, shared pixel boundaries by implementing a straightforward watershed algorithm.

In this work, some steps of the procedure described by Hormann et al. [38] are adopted to implement the aforementioned algorithm fast and accurately. The present work further accounts for the chamber-type attributes of the pores and the corresponding pore identification steps.

### 2.4. Effective Transport Properties

Digital characterization of the material layers was performed in order to derive information on the generated pore network, and to obtain the desired effective properties of the foamy material. These properties were obtained by volume averaging of localized values and are dependent on the physical problem at hand. The mechanism of interest is mass diffusion as well as incompressible (water) flow through the membrane.

#### 2.4.1. Effective Diffusivity

After the reconstructed structure was available, the diffusion equation of the species of interest in the reconstructed domain was solved. According to Fick’s law, the diffusive flux of a chemical species, $\vec{J}$, is

(7) $$\vec{J}=-D_{\mathrm{Bulk}}\nabla c,$$

where $D_{\mathrm{Bulk}}$ is the diffusivity of the species in the carrier fluid. Moreover, the concentration of the species at the inlet, $\;C_{\mathrm{in}}$, and the outlet, $C_{\mathrm{out}}$, boundaries is assumed to be uniform and fixed. The thickness of the simulation domain, $L_{\mathrm{domain}}$, for the present modelling is 7.5 mm. The equation was solved numerically, and the effective diffusion coefficient was extracted by surface averaging of the outgoing flux from the domain, using post-processing of the solution and the tortuosity factor extracted from the following expression:

(8) $$\tau =\frac{\varepsilon }{\left(\frac{D_{\mathrm{eff}}}{D_{\mathrm{Bulk}}}\right)},$$

where *D*_eff_ is the effective diffusivity of the species in the medium,

(9) $$D_{\mathrm{eff}}=\frac{\int \int \vec{J}dA}{A_{\mathrm{tot}}}\frac{L_{\mathrm{domain}}}{C_{\mathrm{out}}-C_{\mathrm{in}}},$$

with *A*_tot_ the surface area of control volume at the outlet patch. In order to perform the calculations required in a fast and simple manner, and to extract the tortuosity factor directly from voxelized micro-structural data, the procedure described in the work of Cooper et al. [39] was used. Specifically, the steady-state diffusion equation with Dirichlet boundary conditions applied on two opposite faces was solved using an advanced finite differences scheme. Namely, an iterative method with over-relaxation was used that considerably accelerates the convergence time by three orders of magnitude, compared to typical direct inversion methods.

#### 2.4.2. Effective Water Permeability Factor

The permeability is calculated using the linear Stokes equations [40,41]. During calculation of the permeability, pressure difference is applied in one of the three principal directions of the 3D reconstructed structure. The pressure difference itself results in a fluid velocity vector field in the void space obeying the following set of PDEs:

(10) $$\eta \nabla^{2}v=\nabla p,$$

(11) ∇⋅v=0,

where v and p are the local velocity and pressure, respectively, and η is the fluid viscosity. Darcy’s equation [41], shown in Equation (12), was then used to calculate the effective permeability of the generated microstructure

(12) $$K_{\mathrm{eff}}=\frac{\eta \langle v\rangle \Delta L}{\Delta P},$$

where 〈v〉 is the volume-averaged local velocity, ΔP and ΔL are the macroscopic pressure difference and the length of the sample domain in the same direction, respectively.

### 2.5. Permeability and Tortuosity Estimation

#### 2.5.1. Permeability Comparison Using Empirical Expressions

Initially, the permeability of the porous medium was compared with semi-analytical expressions found in the literature. The Calmidi empirical correlation [7] was used as one of the bases for performance comparison,

(13) $$K_{c}=0.00073d_{p}^{2}{\left(1-\varepsilon \right)}^{-0.224}{\left(\frac{d_{f}}{d_{p}}\right)}^{-1.11},$$

where *d*_f_*/d*_p_ is the ratio of the ligament thickness to the pore diameter, and is empirically fitted by assuming the G shape factor for the foam branches, estimated by Equation (15):

(14) $$\frac{d_{f}}{d_{p}}=2\sqrt{\frac{1-\varepsilon }{3\pi }}\frac{1}{G},$$

(15) $$G=1-\exp{\left(-\frac{1-\varepsilon }{0.04}\right)}^{}.$$

In addition, the Hooman and Dukhan [42] semi-analytical solution was applied, based οn the hydraulic resistance network, denoted by *K*_HD_:

(16) $$K_{\mathrm{HD}}=0.054d_{P}^{2}\varepsilon \sqrt{1-\varepsilon },$$

Finally, the Depois and Mortensen [43] analytical expression was also used

(17) $$K_{\mathrm{DM}}=\frac{\varepsilon d_{p}^{2}}{4\pi }{\left(\frac{\varepsilon -\varepsilon_{0}}{3\left(1-\varepsilon_{0}\right)}\right)}^{3/2},$$

where *ε*_0_ = 0.64.

#### 2.5.2. Tortuosity Factor from Analytical Expressions

An expression for the tortuosity given from [44,45] following transport path arguments,

(18) $$\frac{1}{\tau_{\mathrm{DP}}}=\frac{3}{4\varepsilon }+\frac{\sqrt{9-8\varepsilon }}{2\varepsilon }\cos\left(\frac{4\pi }{3}+\frac{1}{3}\cos^{-1}\left(\frac{8\varepsilon^{2}-36\varepsilon +27}{{\left(9-8\varepsilon \right)}^{1.5}}\right)\right).$$

The effective transport coefficient for idealized cylinders or spheres embedded in a cubic lattice is given by Equation (19) in the special case of ε_1_ << ε_2_ [46,47], where ε_1_ is the solid phase volume fraction and ε_2_ is the void space volume fraction with $\lambda ={\varepsilon_{1}/\varepsilon }_{2}$, *n* = 2 for spheres and *n* = 1 for cylinders. Using Equation (8), the tortuosity factor τ_R_ can be calculated.

(19) $$\frac{D_{\mathrm{eff}}-D_{2}}{D_{\mathrm{eff}}+nD_{2}}=\lambda \left(\frac{D_{1}-D_{2}}{D_{1}+nD_{2}}\right).$$

#### 2.5.3. Specific Surface Area from Analytical Expressions

An analytical expression that considers an idealized cubic cell model is proposed in [45], which can be further modified to consider a prism instead of a cube, with height equal to the pore size in the longitudinal section. The expression that is obtained depends solely on the porosity and pore diameter of the foam structure, Equation (20).

(20) $$S_{v1}=\frac{2}{d_{p}}{\left(3\pi \left(1-\varepsilon \right)\right)}^{0.5}.$$

An analytical expression for the specific surface area of a foam, which solely depends on porosity, is given by Fourie and Du Plessis [48] and is shown in Equations (21) and (22):

(21) $$S_{v2}=\frac{3}{2d_{p}}{\left(3-x\right)}^{2}\left(x-1\right),$$

(22) $$x=2+2\cos\left(\frac{4\pi }{3}+\frac{1}{3}\cos^{-1}\left(2\varepsilon -1\right)\right).$$

## 3. Results

### 3.1. Extraction of the Cell Diameter

**Figure 6:**
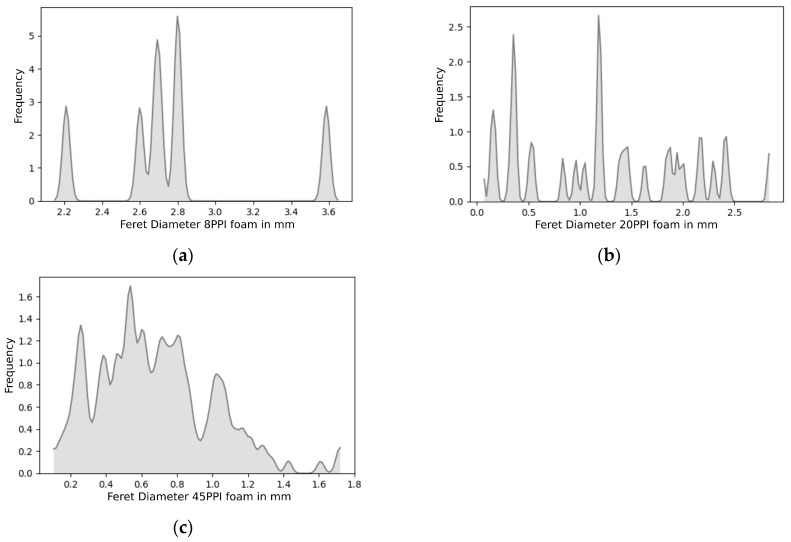
Distribution of the Feret diameter of the spheres used for the reconstruction of the: (**a**) 8 PPI foam, (**b**) 20 PPI foam, and (**c**) 45 PPI foam.

The three processed SEM images representing three different foams were used for further image analysis. Using ImageJ software, as part of the open-source processing package Fiji, we were able to extract the equivalent Feret diameter distribution for the corresponding cells in each image. The Feret diameter distribution is shown in Figure 6 for 8 PPI, 20 PPI, and 45 PPI foams.

The cell size distribution reveals that, for the 8 PPI foam sample, the cell diameters have a more discrete distribution, with 5 major peaks between 2.2 mm and 3.6 mm (Figure 6a). The 20 PPI foam cell distribution is similar to the 8 PPI one. However, it exhibits a wider range of values at which peaks are noted. Finally, the 45 PPI foam sample exhibits a curve that is smoother than the rest. These cell distributions were sampled in order to obtain an arrangement of spheres using the Lubachevsky algorithm, utilizing the corresponding sphere distribution of the sample.

### 3.2. Domain Reconstructions

**Figure 7:**
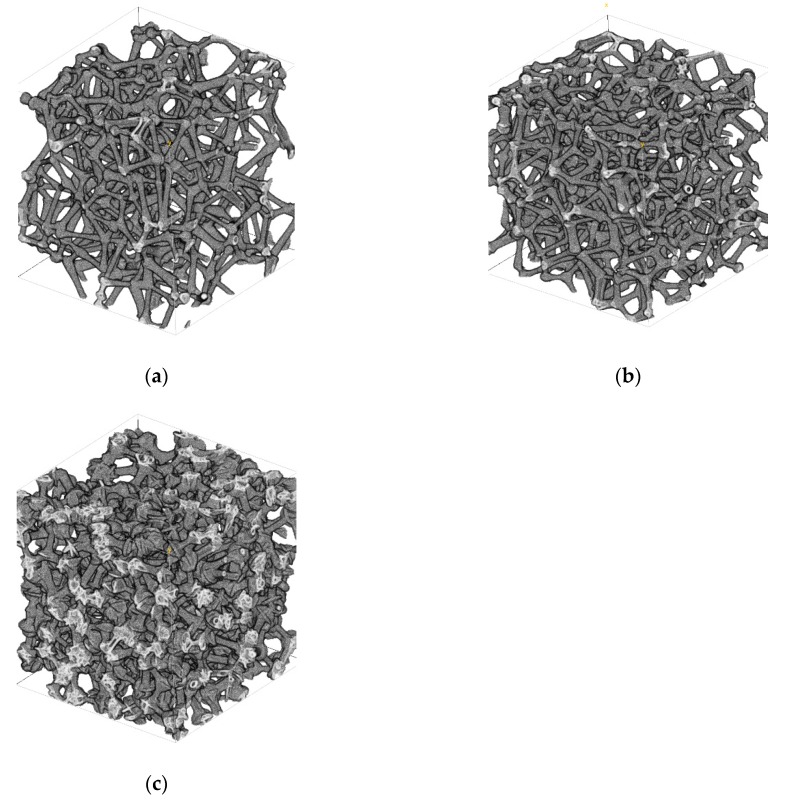
Reconstructed domains with size fixed at 250 × 250 × 250 voxels^3^. (**a**) 8 PPI foam with domain length of 7.5 mm, (**b**) 20 PPI foam with domain length of 7.5 mm, and (**c**) 45 PPI foam with domain length of 4 mm.

Figure 7a–c displays the reconstruction domains obtained for three different foam samples, namely the 8 PPI foam, the 20 PPI foam, and the 45 PPI foam, using the proposed reconstruction algorithm. Qualitatively, the images look similar; nevertheless, different pore densities and branch thicknesses are visible. The average time taken for the reconstructions to be generated on a 12-core Intel^®^ Xeon^®^ E5-2620v2 CPU at 2.10 GHz was 3 ± 0.32 h for 8 PPI foams, 6 ± 0.12 h for 20 PPI foams, and 26 ± 0.03 h for 45 PPI foams. Even though these reconstruction times may appear large in absolute terms, it must be noted that the PPI density affects the reconstruction times. This is due to the fact that an increased cell density leads to an increased number of adjacent cells next to each and every cell—thus, the Laguerre tessellation produces polyhedral cells with an increased number of edges, considerably increasing the computational time of the branch generation and allocation.

### 3.3. Model Validation

The validation of the proposed reconstruction method was done via comparison with experimental data from the literature [30]. A comparison of the experimentally measured porosity of the foam sample and the calculated porosity of the reconstructed foam is shown in Table 1. It is evident that the calculated porosity and the experimental values are in excellent agreement. However, we should note that the experimentally calculated porosity, as well as the characteristic pore radius found in the literature, were used for the estimation of the effective permeability, the specific surface area, as well as the tortuosity factor.

A comparison of the numerically predicted effective permeability on the reconstructions with estimates from analytical expressions from the literature reveals that the proposed reconstruction method provides a substantial improvement in accuracy over previous semi-empirical attempts. Most of the relevant effective permeability expressions found in the literature fail to a large extent to predict the effective permeability with sufficient accuracy, deviating in some cases by one order of magnitude, shown in Table 2.

Furthermore, assuming that the diffusive and the electrical tortuosity factors are identical, the values of the tortuosity obtained from the reconstruction process for each one of the three foamy materials of reference (8 PPI, 20 PPI, 45 PPI) are compared with correlations typical for foamy porous media, shown in Table 3.

It should be noted that the results obtained from the analytical expressions found in the literature are in good agreement with the tortuosity values obtained from the reconstructions. The deviation from the experimental values could be attributed to the different nature of the diffusive tortuosity considered in this study and the electrical tortuosity shown in the experimental results.

In addition to the porosity and the effective transport coefficients used in this study, the surface-to-volume ratio was also of interest. This measurement is acquired by calculating the interfacial area of the control volume, divided by the volume of the control element of the reconstructed domain.

It is evident that the values obtained from the experimental data strongly deviate from the values obtained from literature expressions. However, the values obtained from the reconstruction process are in good agreement with the experimental data, shown in Table 4.

### 3.4. Pore Size Distribution

**Figure 8:**
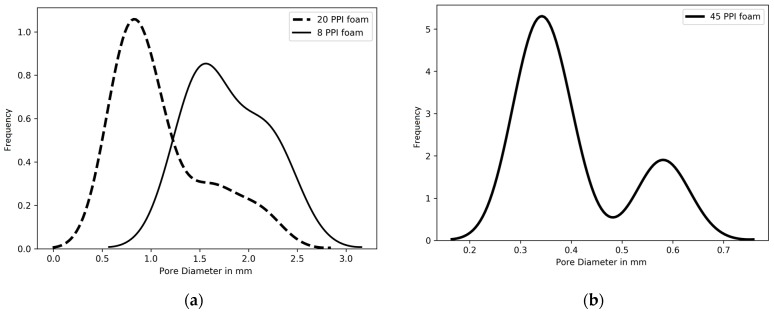
Pore size distribution obtained from the reconstructed images (**a**) 8 PPI and 20 PPI foams, and (**b**) 45 PPI foam.

The PSD of the reconstructed models were computed numerically on the 3D domains using the method of Hormann et al. [38]. The results are presented in Figure 8a for the foams with 8 PPI and 20 PPI, while the 45 PPI foam results are shown in Figure 8b. The average pore diameter of the 8 PPI foam microstructures is 2.01 mm, and pore sizes are within 0.5–3.3 mm for 8 PPI foam with a peak around 1.55 mm; for the 20 PPI foam, the average pore diameter is 1.09 mm, while pore sizes are within 0.05–2.7 mm; for the 45 PPI foam, the average pore diameter is 0.32 mm, which compares favorably to experimental results that are extracted with image processing found in the literature [30]. However, the average pore diameter is a quantitative measurement that is not always a representative ‘unique’ characteristic pore diameter. Moreover, it has been generally accepted that the permeability is related to both the number and size of the pores. This implies that the porosity and the pore size distribution should be evaluated in such a manner that their physical significance is modeled correctly. Thus, in the present work we are evaluating an alternative, direct measurement of the representative pore diameter by utilizing the pore size distributions extracted from the reconstructed domains numerically.

For this reason, laminar permeate circular tubes are considered. Using the notion of the equivalent pore size as described by Reinhardt and Gaber [49], the equivalent pore size for a discrete pore size distribution is directly calculated from Equation (23):

(23) $$r_{e}=\sqrt{\frac{\sum \left(V_{i}r_{i}^{2}\right)}{\sum \left(V_{i}\right)}},$$

where *r*_e_ is the equivalent pore size, *r_i_* is the pore radius of the *i*-th pore, and *V_i_* is the volume occupied by the *i*-th pore. Extraction of the equivalent pore radius for the *i*-th pore provides values different from the expressions found in the literature.

Utilization of the equivalent pore radius offers a notable improvement over the estimation of the permeability predicted by the expressions found in the literature. As can be seen from Table 5, the permeability calculated by Equation (17) or Equation (13) using the new equivalent pore size radius definition still deviates from the experimental value, yet their accuracy is improved. Equation (16) shows a satisfactory agreement with the experimental data with the use of the equivalent pore size radius, especially for the case of the 8 PPI and 20 PPI foam. If the mean diameter, *d*_h_ = 4*ε*/*S*_v,_ is used, closer predictions to the experimental values are noted, Table 6, where *S*_v_ is the specific surface area obtained from image analysis [30].

## 4. Conclusions

This work offers a methodology for improving the description of structure and transport in foamy porous media. A statistical analysis of the SEM images of foams and a stochastic reconstruction of the methodology were presented, which could produce high-precision reconstructions and reliable representations of the foams. The reconstruction process comprises of a Laguerre tessellation performed on a sphere arrangement and a branch placement algorithm. The method is validated against three ceramic foams with different PPI densities. The structures obtained from the reconstruction process were evaluated in terms of porosity, pore size distribution, specific surface area, tortuosity factor, and effective permeability. The results are in good agreement with experimental data found in the literature, while conventional empirical correlations were found to deviate from the actual values of the structural properties of the foams. The extraction of an equivalent pore radius from the reconstructed geometries greatly improved the prediction of the analytical expressions found in the literature for the effective permeability of the foams.

## Figures and Tables

**Table 1:** Foam properties.

Sample	*ε*_rec_	*ε*_exp_ [30]	*d*_p_ (mm) [30]
PPI8	0.938	0.94	2.3
PPI20	0.88	0.88	0.8
PPI45	0.77	0.76	0.36

**Table 2:** Permeability comparison for the corresponding foams.

Sample	*K*_C_ (m^2^)	*K*_HD_ (m^2^)	*K*_DM_ (m^2^)	*K*_SIM_ (m^2^)	*K*_exp_ (m^2^) [30]
PPI8	3.99 × 10^−8^	7.02 × 10^−8^	5.79 × 10^−8^	5.4 × 10^−8^	4.61 × 10^−8^
PPI20	3.7 × 10^−9^	1.05 × 10^−8^	4.69 × 10^−9^	3.28 × 10^−8^	3.22 × 10^−8^
PPI45	4.61 × 10^−10^	2.6 × 10^−9^	3.31 × 10^−10^	7.9 × 10^−9^	8.74 × 10^−9^

**Table 3:** Tortuosity factor results.

Sample	*τ*_DP_	*τ*_R_	*τ*_sim_	*τ*_exp_ [30]
PPI8	1.29	1.036	1.06	1.68
PPI20	1.43	1.088	1.11	1.71
PPI45	1.62	1.28	1.268	1.84

**Table 4:** Specific Surface Area comparison, data in m^2^/m^3^.

Sample	*S*_v1_	*S*_v2_	*S*_v,recon_	*S*_v,exp_ [30]
PPI8	654	562	1526	1680
PPI20	2659	1992	1830	1920
PPI45	8355	4921	2502	2340

**Table 5:** Permeability comparison for the corresponding foams using the equivalent pore diameter, *d*_e_ = 2*r*_e_.

Sample	*d*_e_ (mm)	*K*_C_ (m^2^)	*K*_HD_ (m^2^)	*K*_DM_ (m^2^)	*K*_SIM_ (m^2^)	*K*_exp_ [30]
PPI8	1.88	2.66 × 10^−8^	4.69 × 10^−8^	3.87× 10^−8^	5.4 × 10^−8^	4.61 × 10^−8^
PPI20	1.39	1.11 × 10^−8^	3.18 × 10^−8^	1.41 × 10^−8^	3.28 × 10^−8^	3.22 × 10^−8^
PPI45	0.54	1.03 × 10^−9^	5.86 × 10^−9^	7.46 × 10^−10^	7.9 × 10^−9^	8.74 × 10^−9^

**Table 6:** Permeability comparison for the corresponding foams using the mean diameter, *d*_h_.

Sample	*d*_h_ (mm)	*K*_C_ (m^2^)	*K*_HD_ (m^2^)	*K*_DM_ (m^2^)	*K*_SIM_ (m^2^)	*K*_exp_ [30]
PPI8	2.23	3.97 × 10^−8^	6.22 × 10^−8^	5.48× 10^−8^	5.4 × 10^−8^	4.61 × 10^−8^
PPI20	1.92	2.14 × 10^−8^	6.09 × 10^−8^	2.71 × 10^−8^	3.28 × 10^−8^	3.22 × 10^−8^
PPI45	1.31	6.37 × 10^−9^	3.45 × 10^−9^	4.43 × 10^−10^	7.9 × 10^−9^	8.74 × 10^−9^
